# Performing right upper abdominal cytoreduction: anatomical planes, liver mobilization and occult spaces

**DOI:** 10.1186/s43046-026-00410-6

**Published:** 2026-09-11

**Authors:** M D Ray, Anik Rathee

**Affiliations:** Department of Surgical Oncology, All India Institute of Medical Sciences, New Delhi, India

**Keywords:** Peritoneal Surface Malignancy, CA Ovary, Cytoreductive Surgery, Liver Mobilization, Ray’s Hepato-caval Triangle

## Abstract

**Introduction:**

Optimal Cytoreductive Surgery (CRS) is the cornerstone for curative therapy for advanced ovarian malignancies and other peritoneal surface malignancies (PSMs) and achieving a CC-0 score is the principal prognostic factor for long term survival. The right upper abdomen is one of the most technically demanding regions because disease may be hidden beneath the right liver, along the diaphragm, in Morrison’s pouch, over Glisson’s capsule, and around the hepato-caval groove. Complete liver mobilisation is therefore both an oncologic manoeuvre and a vascular-risk manoeuvre.

**Aims and objective:**

To define and describe the surgical anatomy of the right upper abdomen and discuss the intricacies of dissecting this region.

**Materials and methods:**

This is a retrospective analysis of the prospectively maintained database at Department of Surgical Oncology, Dr BRA-IRCH, All India Institute of Medical Sciences, New Delhi. 205 patients undergoing Cytoreductive Surgery (CRS) with liver mobilization for Ovarian Malignancy were reviewed and perioperative details regarding Upper Abdominal CRS, injuries in the hepato-caval triangle and postoperative complications were recorded.

**Results:**

Among 600 patients undergoing CRS for ovarian malignancies between January 2014 and December 2025, liver mobilization was performed in 205 patients. Most underwent interval CRS after NACT (57.6%). Optimal cytoreduction was achieved in 194 patients (94.6%). RHV injury occurred in 9 patients (4.4%), predominantly during the initial 100 cases.

**Conclusion:**

This article describes a structured approach to right upper abdominal CRS, emphasizing liver mobilisation, exposure of occult spaces, and safe dissection around the hepato-caval junction. Ray’s triangle is presented as a practical danger-zone concept that helps the surgeon understand and avoid RHV-IVC injury during complete mobilisation.

## Introduction

The success of cytoreductive surgery for peritoneal surface malignancy depends not merely on resection of visible bulky disease, but on systematic identification and clearance of all sites of occult peritoneal involvement. Recent literature has reiterated that some of the most consequential failures of CRS occur not because disease is unresectable, but because it remains unseen in hidden recesses, tunnels, and surfaces that are difficult to inspect. The concept of a “pseudo” cytoreduction [1] is particularly relevant in the supracolic compartment, where the right lobe of the liver obscures portions of the diaphragm, Morrison’s pouch, and the hepato-caval region [2].

In this context, complete liver mobilization is not a cosmetic or exposure-only maneuver. It provides a technical rationale when upper abdominal disease is suspected or identified. Classic gynecologic oncology and peritoneal surface malignancy literature has repeatedly emphasized that the right liver must be mobilized medially to expose diaphragmatic and subdiaphragmatic disease, and that the subhepatic and hepato-caval areas may harbor tumor even when the visible diaphragmatic disease burden is limited and imaging modalities are unable to detect them.

The right upper abdomen is uniquely difficult to address surgically because the large right lobe of the liver physically blocks exposure to these critical, gravity-dependent areas.

Consequently, complete liver mobilization—performed by systematically dividing the falciform ligament, followed by the anterior and posterior right coronary ligaments, and finally the right triangular ligament using a medial-to-lateral approach—is essential to reveal occult spaces like Morrison’s pouch and the hepato-caval groove.

Unfortunately, this extensive mobilization carries a severe risk of catastrophic hemorrhage from the right hepatic vein (RHV) and inferior vena cava (IVC) due to traction tears or blind dissection. Ray’s triangle—the anatomical space bounded by the IVC, the acute-angled RHV, and the liver surface—helps conceptualize this danger zone, guiding surgeons to carefully identify these delicate vascular structures before applying lateral traction to the liver.

This duality, oncologic necessity and technical challenge, makes the Hepato-caval Ray’s triangle especially important in CRS. It is a very small triangle above the liver surface where the RHV drains into the IVC at an acute angle. The IVC is covered by a tough sheath called the Hepato-caval ligament (Makuuchi ligament), which is covering this triangle [3]. Anatomical dissection to expose this triangle during separation of the right coronary ligament to separate is a little technical and a little extra force can result in injury to the RHV or IVC at this triangle resulting in catastrophic bleeding. An appreciation of its anatomy helps explain why bleeding during liver mobilization can be sudden and severe, and why safe mobilization demands a deliberate, anatomically informed sequence rather than blind lateral traction.

## Aims and objectives

To describe right upper abdominal cytoreduction as performed in our unit, with emphasis on liver mobilization, occult space clearance, and safe hepato-caval dissection.

### Objectives

#### Primary objective

1. To describe the technical steps of complete liver mobilization during CRS.

#### Secondary objectives

1. To review the surgical anatomy and occult spaces of the right upper abdomen necessary for performing adequate liver mobilization.
2. Retrospective analysis of the patients having undergone Liver Mobilization during CRS at our institute.
3. To present Ray’s triangle as a practical safety concept during dissection near the right hepatic vein and IVC.

## Materials & methods

This is a retrospective analysis of the prospectively maintained database at Department of Surgical Oncology, Dr BRA-IRCH, All India Institute of Medical Sciences, New Delhi. 205 patients who had undergone Cytoreductive Surgery (CRS) and liver mobilization as a part for Ovarian Malignancy at the institute between January 2014 and December 2025 were reviewed and perioperative details regarding Upper Abdominal CRS and Liver mobilization and postoperative complications were recorded. The data was entered and analyzed in Microsoft Excel Version 16.108. Statistical analysis was performed using Welch’s independent samples t-test for continuous variables categorical variables were compared using Fisher’s exact test using IBM SPSS Statistics version 26.0.

### Surgical anatomy of the right upper abdomen

**Fig. 1:**
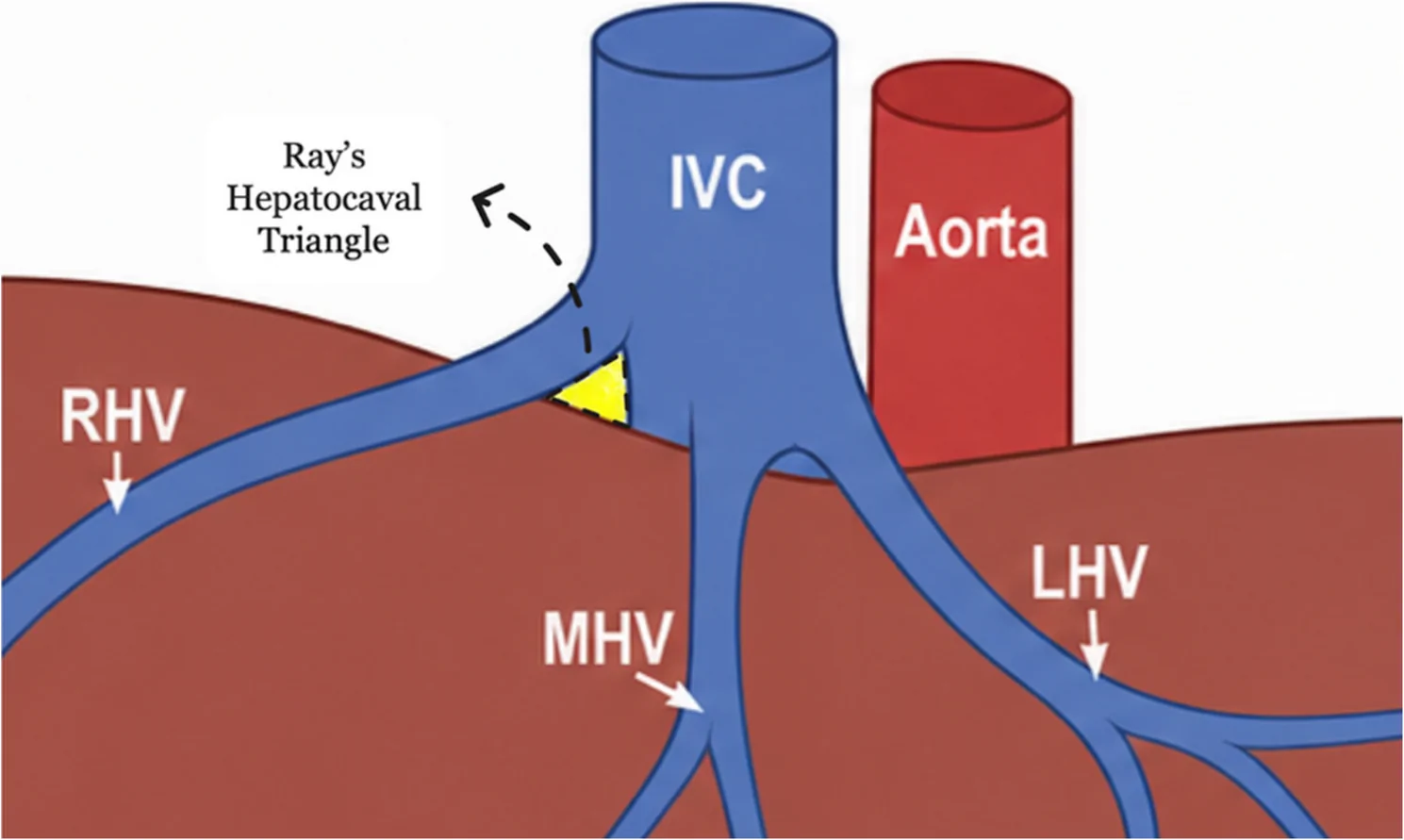
Anterior view of hepato-caval junction and demonstration of Hepato-caval danger zone (Ray’s Triangle) bounded by IVC, RHV and Liver Surface

To perform complete liver mobilization safely, the surgeon must have a meticulous understanding of the attachments of the liver to its surrounding organs and the major central vasculature. The “triangle” in question, the hepato-caval junction, is an acute geometric space bounded superior-medially by the right hepatic vein (RHV), medially and posteriorly by the anterior surface of the inferior vena cava (IVC), and laterally and anteriorly by the bare area of the liver surface (Fig. 1).

### Hepato-caval anatomy and the extra-parenchymatous right hepatic vein

The RHV is the largest vein in the hepatic venous system and dictates profound changes in the venous architecture of the liver. Dissection studies on fresh cadaveric subjects have shown that the RHV joins the IVC at a distinctly acute angle, measuring on average 28.6° ± 5.6° (range 20°–35°). This acute angle forms the apex of the vulnerable triangle. Furthermore, the extra-parenchymatous segment of the RHV, the length between its exit from the hepatic parenchyma and its entry into the IVC, is remarkably short, measuring an average of only 8.6 ± 1.8 mm (range 6–12 mm). In the majority of cases (approximately 70%), this short segment is devoid of collateral branches, but in 30% of cases, it is joined by a branch close to its exit point from the parenchyma [3]. 

The Makuuchi Ligament: The hepato-caval junction is not freely exposed; it is obscured by fibrous connective tissue of varying thickness. This tissue forms what is clinically known as the “vena cava ligament” or “Makuuchi ligament”. This ligament physically obstructs the direct view of the hepato-caval junction, making the isolation of the extra-parenchymatous RHV difficult and highly hazardous if approached blindly. Primary division of this ligament is an essential step to safely access the triangular space and facilitate the dissection of the RHV [3]. 

Accessory Right Hepatic Veins Anatomy in this region is further complicated by the presence of accessory right hepatic veins (also known as posterior-inferior right hepatic veins). During the release of the right edge of the IVC, one or multiple accessory veins are identified in up to 90% of cases. While most of these veins have a narrow diameter (< 0.5 cm), in roughly 10% to 30% of cases, there is a large posterior-inferior accessory vein that is greater in diameter than the superior RHV itself, draining the entirety of segment VI and parts of segment VII. The presence of these accessory veins traversing the lower portion of our anatomical triangle presents an immediate bleeding risk during the mobilization of the right hepatic lobe.

### Need for complete liver mobilization

Despite advances in preoperative imaging such as contrast-enhanced CT and MRI, the detection of miliary or plaque-like peritoneal disease remains challenging, and imaging often underestimates the true extent of peritoneal carcinomatosis. Consequently, surgeons rely heavily on visual inspection during CRS. Computed tomography has limited sensitivity for small peritoneal deposits; Koh et al. reported a sensitivity of only 11% for detecting peritoneal lesions smaller than 5 mm [4]. 

Liver mobilization during upper abdominal CRS should be understood as a technical maneuver for exposure, assessment, and safe peritonectomy rather than as an independent oncological intervention. Its extent depends on tumor burden, adhesions, and the need to expose occult right upper abdominal spaces. In patients with macroscopic disease in the right upper quadrant, complete mobilization of the right lobe is recommended to expose the right subphrenic space, Morrison’s pouch, Glisson’s capsule, retro-hepatic IVC, and the hepato-caval junction. As noted by the PSOGI-ESGO-ISSPP Lyon consensus, the right lobe of the liver must be completely mobilized by dividing all its attachments (the right coronary and triangular ligaments) and the falciform ligament in the presence of upper quadrant disease.

In the present cohort, liver mobilization was performed for four indications: routine mobilization when retraction alone did not provide adequate exposure; tumor-driven mobilization when disease was suspected or identified in the right subphrenic region, Morrison’s pouch, Glisson’s capsule, porta hepatis, or hepato-caval region; exposure for assessment of occult spaces not visible on limited inspection; and facilitation of peritonectomy, particularly for right diaphragmatic, subhepatic, and retro-hepatic peritoneal clearance. Failure to expose these regions may lead to what is termed a “pseudo” cytoreduction [1], as residual cancer nodules are left behind in occult peritoneal spaces [5]. As Sugarbaker famously stated, “It’s what the surgeon doesn’t see that kills the patient“ [6].

### Occult spaces exposed by liver mobilization

Complete liver mobilization is important to expose hidden disease in the right upper abdomen. Without this step, residual deposits may be missed behind the liver, beneath the diaphragm, or along the hepato-caval region.

#### Right subphrenic space

Disease under the right hemidiaphragm may be hidden by the liver, especially when deposits are plaque-like or miliary. Mobilization allows direct inspection and safe diaphragmatic stripping or resection.

#### Morrison’s pouch

Disease in the hepatorenal recess is often missed unless the liver is fully mobilized. Exposure should include the right kidney, adrenal plane and posterior abdominal wall.

#### Hepato-caval groove

This is a critical occult space where disease may lie between the posterior liver and IVC. Clearance here links oncologic cytoreduction with the vascular safety concept of hepato-caval danger zone (Ray’s triangle).

#### Glisson’s capsule and liver fissures

Superficial liver deposits may be treated by capsulectomy or electro-evaporation. The liver surface and fissures should be inspected after mobilization to avoid missed deposits.

#### Foramen of winslow

The foramen of Winslow and posterior hepatoduodenal ligament should be inspected as adjacent occult spaces, particularly when disease is present in the subhepatic, periportal or lesser sac region.

The hepato-caval groove is the most surgically critical of these spaces because clearance brings the surgeon close to the right hepatic vein–IVC junction. This is where hepato-caval danger zone (Ray’s triangle) acts as a practical danger-zone concept during complete liver mobilization.

### The hepato-caval danger zone (Ray’s triangle)

**Fig. 2:**
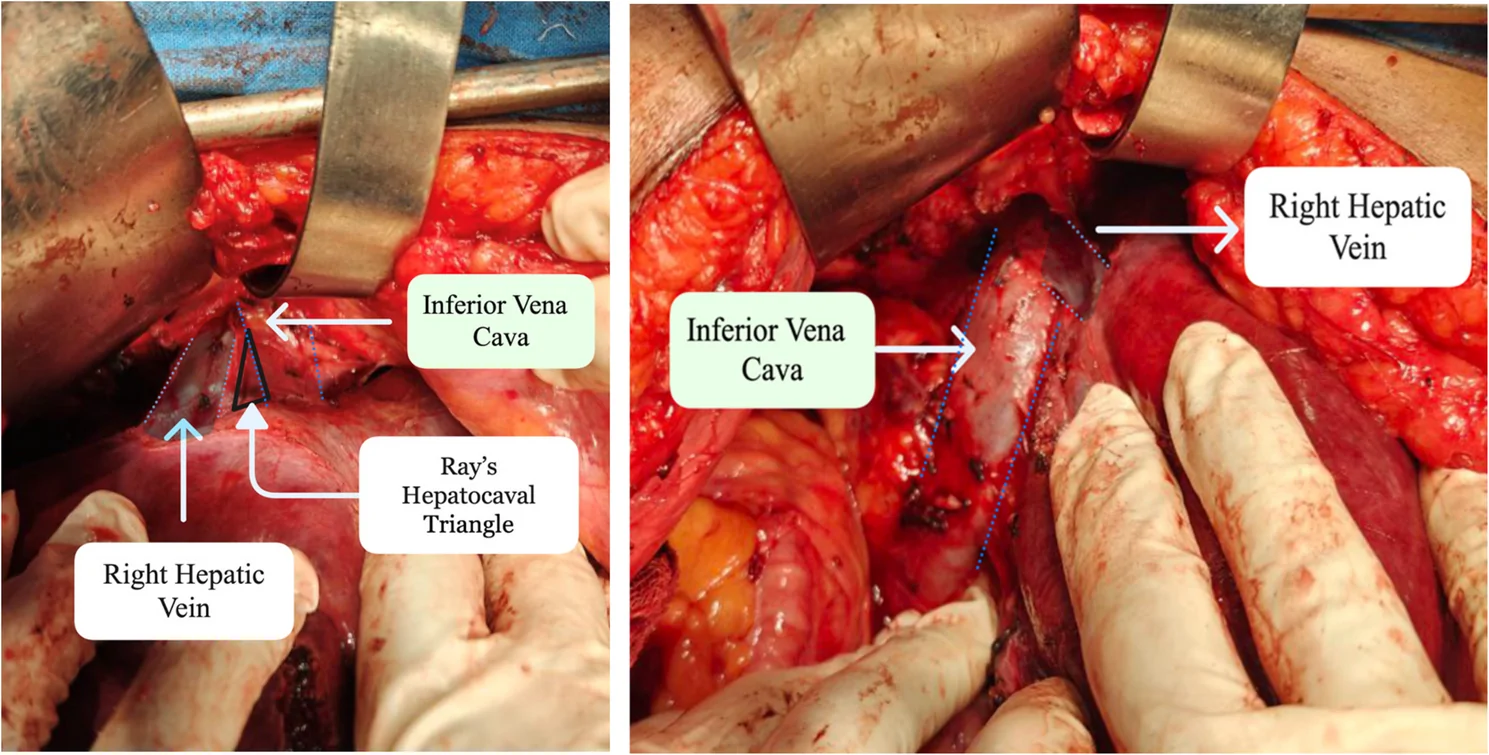
Intraoperative image post complete liver mobilization showing Acute Hepato-caval Angle and hepato-caval danger zone (Ray’s triangle) bounded by IVC, RHV and liver surface. The second image shows the IVC-RHV junction with the liver reflected medially

The proposed “Ray’s triangle” is the hepato-caval danger zone bounded by medially - right wall of the suprahepatic IVC, laterally - acute angled RHV, inferiorly - superior surface of the liver, and anteriorly – Hepato-caval ligament. This space is not a classical anatomical triangle, rather a surgical high-risk zone where any injury can have catastrophic consequences. The author observes that many surgeons avoid complete liver mobilization because of the fear of right hepatic vein injury, which can lead to catastrophic hemorrhage and major perioperative morbidity. A structured anatomical approach with clear identification of the hepato-caval danger zone may reduce this risk and improve surgical confidence (Fig. 2).

### Why the hepato-caval junction is vulnerable?

Traction Injury: Because the right hepatic vein is extraordinarily short (averaging 8.6 mm) and joins the IVC at an acute angle. Any excessive or forceful medial or downward traction on the right lobe of the liver during mobilization can easily avulse the RHV from the IVC.

Blind Dissection: The presence of the dense Makuuchi (vena cava) ligament means that the RHV is often hidden. Surgeons attempting to encircle or clear the IVC without first sharply dividing this fibrous tissue may blindly puncture the thin-walled RHV or the anterior wall of the IVC.

Accessory Vein Avulsion: As the surgeon mobilizes the liver by dissecting the right triangular and coronary ligaments, the right retro-hepatic edge of the IVC is exposed.

Tumor Infiltration: In advanced peritoneal malignancies, the tumor deposits often infiltrate the diaphragmatic muscle and can extend right up to the lateral border of the IVC. Stripping the peritoneum in the acute angle between the liver and the IVC can easily result in entering the vessel lumen if the anatomical planes are distorted by tumor or post-chemotherapy fibrosis.

Consequently, a tear at the hepato-caval junction or RHV or IVC results in high volume, low pressure venous bleeding the floods the surgical field instantly, with sudden hemodynamic compromise of the patient.

Hence, an inexperienced surgeon may avoid complete dissection in this area which is prone to have residual or recurrent disease.

### Techniques to mitigate injury during liver mobilization

- The Medial-to-Lateral Approach: As highlighted in laparoscopic and open techniques, dissecting the superior coronary and left triangular ligaments after identifying the suprahepatic IVC provides superior exposure. Initiating the dissection blindly from the lateral aspect inward risks injuring unseen diaphragm vessels or collateral veins traversing this area.
- Division of the Vena Cava Ligament: Primary sectioning of the fibrous Makuuchi ligament overlying the hepato-caval junction is mandatory. This exposes the 5 to 10 mm extra-parenchymatous segment of the RHV, allowing the surgeon to clearly visualize the acute angle and protect the vein.
- Vascular Control (Hepatic Vascular Exclusion): In selected cases with bulky upper abdominal disease abutting the hepato-caval junction, prior vascular control of the right hepatic vein may be considered as a potential safety maneuver. Peschaud et al. demonstrated that the extra-parenchymatous segment of the right hepatic vein is anatomically clampable in most cases; in their study, the terminal extra-parenchymatous RHV was free of collateral branches in 70% of cases, with a mean clampable length of 8.6 ± 1.8 mm. Selective extrahepatic RHV clamping may theoretically reduce hepatic venous back-bleeding and may avoid the hemodynamic consequences of total hepatic vascular exclusion. However, this maneuver was not routinely used or evaluated in the present cohort and is therefore discussed only as a possible vascular-control strategy for selected high-risk hepato-caval dissections.
- Preservation of the Areolar Tissue: When stripping the peritoneum off the diaphragm and reflecting it onto the liver, the loose areolar tissue surrounding the IVC must be preserved. The tumor rarely encases the IVC or RHV directly, and remaining in the areolar plane prevents vascular injury.
- During suprahepatic IVC and right hepatic vein dissection, maintaining a low central venous pressure (CVP), when physiologically tolerated, can reduce hepatic venous back-bleeding and improve visualization of the hepato-caval danger zone. This requires close communication with the anesthesia team, particularly during right liver mobilization, division of the coronary and triangular ligaments, and exposure of the retro-hepatic IVC. The strategy should be individualized to avoid hypovolemia, hypotension, renal hypoperfusion, or hemodynamic instability.

All procedures in the present cohort were performed by an open approach through a midline laparotomy. After adequate exposure of the upper abdomen, the first step was mobilization of the liver to access the right subphrenic and subhepatic spaces. The falciform ligament was divided, followed by careful division of the right coronary and triangular ligaments. The liver was then mobilized medially to expose the retro-hepatic inferior vena cava. Once the IVC was clearly visualized, dissection was continued cranially to identify the right hepatic vein and its relationship with the suprahepatic IVC.

Particular care was taken during dissection in the hepato-caval danger zone, where the right hepatic vein, inferior vena cava, and posterior surface of the right liver are in proximity. In this region, dissection was performed using fine, controlled movements under direct vision, avoiding blind traction or forceful separation of adhesions. Accessory hepatic veins and short hepatic venous tributaries encountered along the retro-hepatic IVC were carefully identified by fine retro-hepatic dissection and preserved whenever feasible. When preservation was not possible because of direct tumor involvement or bleeding risk, vascular control was obtained before division.

In cases with adhesions or tumor infiltration, the dissection was individualized according to the operative findings. Post-neoadjuvant chemotherapy fibrosis occasionally obscured the normal anatomical planes, particularly in the retro-hepatic region and Morrison’s pouch. In two patients, dense fibrosis in this region prevented safe identification of the dissection planes, and further attempt at complete clearance was abandoned to avoid major vascular injury. These cases were therefore included among patients with incomplete cytoreduction.

## Results

At our center, 600 patients were operated for CRS between January 2014 and December 2025 for ovarian malignancies and 205 patients underwent liver mobilization, demographics of which have been shown in the Table 1.

**Table 1:** Demographics Data of the patients

Variable	Number of Patients
Mean Age	48.6 *±* 12.60 years
Performance Status	
ECOG – 0	
ECOG – 1	132 (64.39%)
ECOG – 2	25 (12.19%)
Comorbidities, (any)	92 (44.87%)
Histology	
Low Grade Serous Ovarian Carcinoma	59 (28.78%)
High Grade Serous Ovarian Carcinoma	146 (71.22%)
FIGO Staging	
Stage II	23 (11.2%)
Stage III	159 (77.6%)
Stage IV	23 (11.2%)
Received Neoadjuvant Therapy	118 (57.56%)
Operation Setting	
Upfront	87 (42.44%)
Interval	118 (57.56%)
Mean PCI	8.9 *±* 4.44
Total CC Score	
CC-0	178 (86.83%)
CC-1	16 (7.69%)
CC-2	11 (5.36%)

All the patients underwent cytoreduction by open technique, and among 205 patients requiring liver mobilization, right subdiaphragmatic peritonectomy was performed in all patients. Full-thickness diaphragmatic resection was required in 87 patients (42.44%), reflecting extensive diaphragmatic involvement. Morrison’s pouch clearance was performed in 139 patients (67.8%), while Glisson’s capsulectomy was needed in 46 patients (22.44%) for liver surface disease. (Table 2) Additional upper abdominal procedures included periportal clearance in 27 patients (13.17%) and splenectomy in 38 patients (18.53%), indicating the frequent need for multi-visceral upper abdominal cytoreduction.

**Table 2:** Procedures and their frequencies done as a part of upper abdominal cytoreduction

Upper Abdominal Procedures Performed	Number (*n* = 205)
Right subdiaphragmatic Peritonectomy	205 (100%)
Full Thickness Diaphragm Resection	87 (42.44%)
Morrison’s pouch clearance	139 (67.8%)
Glisson’s Capsulectomy	46 (22.44%)
Periportal Clearance	27 (13.17%)
Splenectomy	38 (18.53%)

Out of these patients, 87 were operated in the upfront setting, and 118 had undergone Neoadjuvant Chemotherapy (NACT) and were operated in the interval setting. Mean age for the cohort was 48.6 years *±* 12.60 years (SD). Mean Peritoneal Carcinomatosis Index (PCI) observed in our cohort was 8.9 *±* 4.44 with optimal cytoreduction being achieved in 194 patients (CC-0 in 178 and CC-1 in 16 patients) with liver mobilization being done in all patients to access the Hepatorenal Pouch and Subdiaphragmatic peritoneum adequately.

11 patients underwent CC-2 resection and the reasons for incomplete cytoreduction were reviewed and categorized according to the intraoperative limiting factor. 4 patients had unresectable disease involving the porta hepatis. 3 patients had poor intraoperative physiological tolerance, precluding continuation of extensive cytoreductive procedures. 2 patients had disease involving the root of the superior mesenteric artery, where complete clearance was not feasible without unacceptable vascular risk. In the remaining 2 patients, dense fibrosis in the region of Morrison’s pouch obscured the normal anatomical planes, making safe dissection and complete clearance difficult. Thus, the CC-2 resections primarily reflected disease at anatomically critical sites or patient-safety considerations rather than isolated failure of right upper abdominal clearance.

**Table 3:** Complications and consequences of injuries during liver mobilization

Variable	RHV injury (*n* = 9)	No RHV injury (*n* = 196)	*p*- value (Confidence Intervals)
Intraoperative Blood Loss	1292 ml *±* 183 ml	897 *±* 159 ml	0.00016 (253.5–536.5 ml)
Postoperative ICU Stay	2.14 *±* 0.9 days	1.3 *±* 0.5 days	0.023 (0.15–1.53 days)
Clavien-Dindo Grade III and above	2 patients (22.2%)	20 patients (10.2%)	0.249 (4.7% − 44.7%)
Re-exploration	None	2 patients (1%)	-
Pulmonary Complications	3 patients (33.3%)	25 patients (12.8%)	0.109 (1.4% − 52.1%)
Total Hospital Stay	7.57 *±* 2.1 days	6.23 *±* 1.6 days	0.094 (0.28–2.96 days)

RHV injury occurred in 9 of 205 patients, corresponding to an overall incidence of 4.4% (95% CI, 2.3–8.1%). An observed reduction in RHV injury rate was noted between the early and later parts of the cohort, from 7.0% (7/100; 95% CI, 3.4–13.7%) to 1.9% (2/105; 95% CI, 0.5–6.7%). This difference did not reach statistical significance using Fisher’s exact test (*p* = 0.095), and should therefore be interpreted as an observed temporal reduction rather than definitive evidence of a causal learning-curve effect.

Patients with RHV injury had significantly higher intraoperative blood loss compared with patients without RHV injury, with a mean difference of 395 ml, 95% CI 253.5–536.5 ml; *p* = 0.00016. Postoperative ICU stay was also significantly longer in the RHV injury group, with a mean difference of 0.84 days, 95% CI 0.15–1.53 days; *p* = 0.023. There were no statistically significant differences in Clavien–Dindo grade III or higher complications, re-exploration, pulmonary complications, or total hospital stay between the two groups (Table 3).

## Discussion

Optimal CRS that achieves CC-0 is the principal prognostic factor that dictates long term survival in advanced epithelial ovarian malignancies as 60–70% patients have supra-mesocolic disease [7]. Hence, complete liver mobilization occupies a distinctive place in upper abdominal cytoreductive surgery because it serves simultaneously as an exposure maneuver, an oncological maneuver, and a potentially hazardous vascular maneuver. These functions are closely interrelated and cannot be considered independently. Liver mobilization places the surgeon in close proximity to one of the most technically demanding venous regions in abdominal surgery [3, 8].

Failure to systematically divide the hepatic ligamentous attachments to completely mobilize the liver may lead to what is termed a “pseudo-cytoreduction”. In this scenario, the operative field might appear macroscopically clear, but residual cancer nodules are left behind in unseen recesses. As Sugarbaker famously noted, “It’s what the surgeon doesn’t see that kills the patient”. Thus, complete liver mobilization is a technical maneuver required to transition a pseudo-complete cytoreduction into an optimal clearance [1]. 

The anatomical basis for the risk of liver mobilization has been clearly demonstrated by Peschaud et al., who quantified the extra-parenchymal anatomy of the right hepatic vein. Their cadaveric study showed that the RHV enters the inferior vena cava at an acute angle and that the mean length available for extrahepatic control is only 8.6 mm [3]. They also documented the frequent presence of accessory right hepatic veins and described fibrous tissue at the hepato-caval junction, including the so-called Makuuchi ligament, which may obscure direct access to the venous confluence [3]. These findings are highly relevant in operative practice. The RHV is not simply short; it is short in a mechanically vulnerable configuration, such that traction on the right lobe during mobilization can be directly transmitted to its junction with the IVC.

In our series, RHV injury occurred in 9 patients and, although all were successfully repaired, these events were associated with greater blood loss and longer ICU and hospital stay, underscoring their clinical significance. These findings reinforce that complete liver mobilization should be undertaken as an anatomically disciplined vascular dissection, with meticulous understanding of the hepato-caval triangle, careful medial-to-lateral release, and controlled traction to minimize preventable venous injury while attempting to preserve oncologic completeness.

From a technical perspective, the literature suggests that vascular safety in this region is largely determined by operative sequence and maintenance of the correct dissection plane.

The oncologic rationale for accepting these risks is supported by recent work on occult peritoneal spaces. Glehen, Bhatt, and Sugarbaker have emphasized that complete cytoreductive surgery depends not only on removal of obvious disease, but also on deliberate exploration of anatomically concealed spaces that are not visible on routine inspection [2]. Therefore, incomplete mobilization of the liver may create a false sense of operative adequacy. In this setting, liver mobilization should not be considered an optional adjunct to right upper quadrant peritonectomy, but rather an integral component of a complete oncologic clearance [2, 7, 9].

The reports by Shin et al. and Kehoe et al. complement this technical understanding by underscoring both the operative and physiological consequences of liver mobilization [9, 10]. Both emphasize the need for careful exposure of the central vasculature beneath the coronary ligament reflections. Shin et al. additionally observed that forceful leftward retraction of the liver may transiently compress the IVC and result in hemodynamic instability, highlighting that safe mobilization requires not only precise dissection but also measured retraction and close communication with the anesthetic team [9]. Kehoe et al. further reinforce that diaphragmatic surgery in this region must be systematic, including formal assessment of diaphragmatic integrity and repair when full-thickness entry has occurred [10].

Safely negotiating hepato-caval danger zone (Ray’s triangle) involves a significant surgical learning curve. In our institutional experience of 600 patients operated for CRS for ovarian malignancies, 205 patients required complete liver mobilization. RHV injuries occurred in 9 of these patients (4.4%), which resulted in sudden high-volume bleeding, significantly increased intraoperative blood loss, and prolonged intensive care and overall hospital stays. Notably, 7 of these 9 injuries occurred during the first 100 cases, compared to only 2 injuries in the subsequent 105 patients. This demonstrates a clear learning curve and reinforces the fact that vascular safety in the right upper abdomen is achieved through anatomical discipline, primary division of the Makuuchi ligament, and measured medial-to-lateral release of attachments, rather than relying on blind lateral traction.

From an academic standpoint, the hepato-caval triangle may be regarded as a surgically constructed anatomical concept describing the high-risk zone formed by the RHV, the adjacent retro-hepatic or suprahepatic IVC, and the posterior-superior surface of the right hepatic lobe near its ligamentous reflections. Its value lies not in its status as a formally recognized classical space, but in its practical capacity to localize the point where several factors associated with operative difficulty converge: a short extra-parenchymal venous segment, acute venous angulation, frequent accessory venous anatomy, obscuring fibrous tissue, and the need for organ rotation during dissection. Thus, the hepato-caval triangle is best understood not as a discrete embryological or fascial compartment, but as a procedurally relevant danger zone whose recognition may improve anatomical orientation during liver mobilization and right upper quadrant cytoreduction [2, 3, 7].

Overall, the available literature supports a consistent interpretation. Complete liver mobilization is justified when right upper quadrant disease is present because it enables exposure of occult peritoneal spaces and thereby contributes to a genuinely complete cytoreduction. However, this maneuver should be approached not as routine ligament division, but as a controlled vascular dissection in the region of hepato-caval triangle. The operative principle is therefore not aggressiveness alone, but anatomically disciplined mobilization: early identification of the hepato-caval junction, controlled medial-to-lateral release of attachments, preservation of the correct areolar plane, and deliberate inspection of the hepato-caval and subhepatic recesses once exposure has been achieved [2, 10]. 

## Conclusion

Right upper abdominal cytoreduction requires more than removal of visible diaphragmatic disease. It demands complete mobilization of the liver, systematic inspection of hidden peritoneal recesses, clearance of Morrison’s pouch, the hepato-caval groove, Glisson’s capsule and related occult spaces, and safe navigation of the RHV-IVC junction. Ray’s triangle provides a practical anatomical warning zone during this maneuver. In experienced hands, respecting these planes converts liver mobilization from a feared vascular step into a reproducible technical maneuver that improves the likelihood of complete cytoreduction.

## Data Availability

No datasets were generated or analysed during the current study.
